# Children in the Cotton Trade

**Published:** 1920-04-03

**Authors:** 


THE PUBLIC WELL-BEING—(*continued*)

### CHILDREN IN THE COTTON TRADE.

### What Legislation will soon Accomplish.

With the coming of the Industrial Revolution of the eighteenth century began the association of children with factory work—an association marred at the outset by heedlessness and cruelty, but, on the whole, tending towards humane conditions of employment. Even to-day much remains to be done; and there are many who think that the final act of justice to the children will only be accomplished by their removal altogether from industrial work. That will be accomplished when the Education Act of 1918, forbidding the employment of "half-timers," comes fully into operation.

Oldham is a typical cotton town, and the figures just published by its juvenile employment officer help one to understand the conditions that prevail pretty generally throughout the industry. The period under review covers the three months November, December, January. During those months, of the 297 boys who left school, no fewer than 231 had been "half-timers"—that is to say, they had spent half of the day at school and half at work in the mill. Of 343 girls who left school in the same period 218 had been half-timers. Taking these 297 boys, the figures show that 64 per cent. of them (188) continue to work in the textile industry, 70 go to work at trades, 37 take up "other occupations," and 2 remain at home. In the case of the 343 girls, 283 remain in the textile industry (82.5 per cent.), 14 enter trades, 24 take up "other occupations," and 22 remain at home. The educational standard which the boys had reached when they left school is shown in the following table:—

| | Half-timers. | Non half-timers. |
|---|---|---|
| Standard Ex 7 ... | 24 | 6 |
| Standard 7 ... | 69 | 26 |
| Standard 6 ... | 68 | 20 |
| Standard 5 ... | 37 | 8 |
| Standard 4 ... | 6 | 3 |
| Standard 3 ... | 3 | 2 |
| Secondary school | — | 1 |

In the case of the girls the standard was as follows:—

| | Half-timers. | Non-half-timers. |
|---|---|---|
| Standard Ex 7 ... | 15 | 6 |
| Standard 7 ... | 94 | 47 |
| Standard 6 ... | 71 | 41 |
| Standard 5 ... | 30 | 20 |
| Standard 4 ... | 8 | 8 |
| Standard 3 ... | — | 2 |
| Special school ... | — | 1 |

It would appear thus that educationally the half-timer reaches as high a standard as the child whose whole day is spent at school. In fact, the percentages on the above figures are rather in favour of the half-timers in the case of boys, but with girls the balance is the other way.

**THE PUBLIC WELL-BEING—*(continued)*.**

The number of children employed on "half-time" has increased since the war. The wages now paid are higher than they have ever been; and this, coupled with the high cost of living, which has influence with many parents in deciding whether their children shall work or not, has led to a great increase in "half-timers."

The children spend their time at the mill in the morning and afternoon during alternate weeks. In the spinning department work generally begins for the children, during the morning week, at 7.45 A.M. and goes on until 12.15, the afternoons being devoted to school. The following week the child attends school in the morning, and begins work at the mill at 1.15 P.M., leaving off at 5.30. His earnings will average about 20s. a week. The boy is not directly employed by the owner of the mill. The unit of employment in a spinning mill is four spinning "mules," and the employer pays the spinner (an adult workman) on the basis of every hundred pounds of yarn produced. Out of the sum received, the spinner pays the boys who assist him. The sum varies in different districts, and though, as a rule, there is no definite amount laid down, there is an unwritten law on the subject which secures a pretty uniform rate of pay.

Each spinner customarily employs two boys, who are known as piecers, because part of their work is to piece together threads which are broken in the process of spinning. They also act in any general capacity the spinner desires—sweeping and fetching and carrying. The sharper boys pick up the trade of the spinner, and so there is a constant supply of labour to this branch of the cotton industry. Indeed, as each spinner employs, as a rule, two piecers, there is an over-supply, and, as the figures from Oldham show, many "half-timers" have to look to other trades for employment when their school days are over. In the weaving processes of cotton manufacture the conditions in this respect are practically the same as in spinning, the weaver employing and paying his juvenile assistants, many of whom, when their "half-time" days are over, become weavers on their own account.

The conditions in which this child-labour is employed are, necessarily, not ideal conditions for children to be in, despite the many improvements which recent years have seen, due either to the humanity of employers or the demands of the Factory Acts. The question that really has to be settled is not whether conditions in the mills can be improved, but whether it is right that children should, in any circumstances, be forced into industry at so early an age. With all the protection of machinery that has been introduced, with all the improvements in ventilation and general sanitary arrangements, there yet remains, and always will remain, while the system lasts, the loss of opportunity for intellectual improvements, the loss of resiliency that a child's mind should have, the compelling of thought and effort at too early an age along defined lines. Attendance upon a machine, however well protected the machine may be, and housed in however commendable a factory, is not an occupation to give play to the free thought of a child. For years the necessities of parents have conspired with the desires of employers to keep the system in being. Only a legislative command, such as that contained in Mr. Fisher's Education Act, can end it. There are many leaders, both of masters and men, who point to themselves as products of the half-time system, and ask how, if it has produced such splendid fellows, it can be wrong? That they should ask the question implies that they are not so splendid as they think, for they seem unable to see that they have succeeded in spite of the system and not because of it.

